# Antegrade recanalization of parent artery in proximal occlusion of distal posterior inferior cerebellar artery ruptured aneurysm: A case report

**DOI:** 10.1097/MD.0000000000028260

**Published:** 2021-12-17

**Authors:** Hyung-Gyu Jang, Jung-Soo Park

**Affiliations:** a Department of Neurosurgery, Jeonbuk National University Medical School and Hospital, Jeonju, South Korea; b Research Institute of Clinical Medicine of Jeonbuk National University - Biomedical Research Institute of Jeonbuk National University Hospital, Jeonju, South Korea.

**Keywords:** aneurysm, angiography, posterior inferior cerebellar artery

## Abstract

**Rationale:**

Distal posterior inferior cerebellar artery (PICA) aneurysms are extremely rare. Herein, we describe a case of PICA pseudoaneurysm with proximal occlusion achieved using detachable coils, but antegrade recanalization, which showed a normal PICA configuration on follow-up angiography. Possible mechanisms of the recanalization and lesions are also discussed.

**Patient concerns:**

The patient was an 80-year-old woman with a subarachnoid hemorrhage (SAH) resulting from a distal PICA-ruptured aneurysm, initially misdiagnosed as a non-aneurysmal traumatic SAH.

**Diagnosis:**

On hospitalization day 10, the patient developed rebleeding, and brain computed tomography angiography confirmed a distal PICA pseudoaneurysm.

**Intervention:**

Endovascular coil embolization was performed. Inevitably, the proximal PICA was occluded using detachable coils, and complete occlusion of the affected PICA was confirmed on the final angiogram.

**Outcome:**

Fortunately, the patient recovered fully without any neurological sequelae. One year after the procedure, a follow-up angiography was performed, which revealed recanalization of the previously occluded PICA, with normal configuration and no visible aneurysmal dilatation.

**Conclusion:**

Even if the SAH is scanty and predominantly in the perimesencephalic cistern, performing a catheter-based angiography is essential. In the case of proximal occlusion of the parent artery without internal trapping in endovascular treatment of PICA pseudoaneurysm, follow-up examination with a short-term angiography might be crucial.

## 1 Introduction

Posterior inferior cerebellar artery (PICA) aneurysms are relatively uncommon vascular lesions, accounting for approximately 0.5% to 3% of all intracranial aneurysms.^[1]^ In patients with PICA aneurysm, rebleeding can occur easily after an acute period of first-time rupture, resulting in a fatal course. The majority of PICA aneurysms arise from the proximal PICA (PICA–vertebral artery junction), with distal PICA aneurysms being extremely rare.^[2]^ Distal PICA aneurysms are easily missed by clinicians, leading to misdiagnosis and delayed treatment. Herein, we report a case of delayed PICA pseudoaneurysm that was treated with proximal occlusion using detachable coils, but antegrade recanalization, which showed a normal PICA configuration on follow-up angiography. We discuss the possible mechanism of recanalization of the occluded PICA and reflect on the experiences from this case.

## 2 Consent for publication

Written informed consent was obtained from the patient for publication of this case report and accompanying images. This case report was conducted under the Declaration of Helsinki.

## 3 Case report

**Figure 1:**
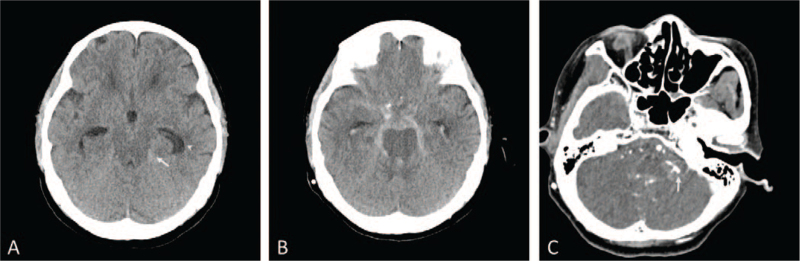
(A) Initial non-contrast brain computed tomography (CT) scan showed a scanty subarachnoid hemorrhage (SAH) on left ambient cistern (arrow) with enlarged ventricle (arrow head). (B) The brain CT taken at 10th hospital day revealed SAH on all cistern with intraventricular hemorrhage. (C) The thin axial source images of CT angiography taken at 10th hospital day showed a suspicious lesion of aneurysm on distal posterior inferior cerebellar artery (arrow).

**Figure 2:**
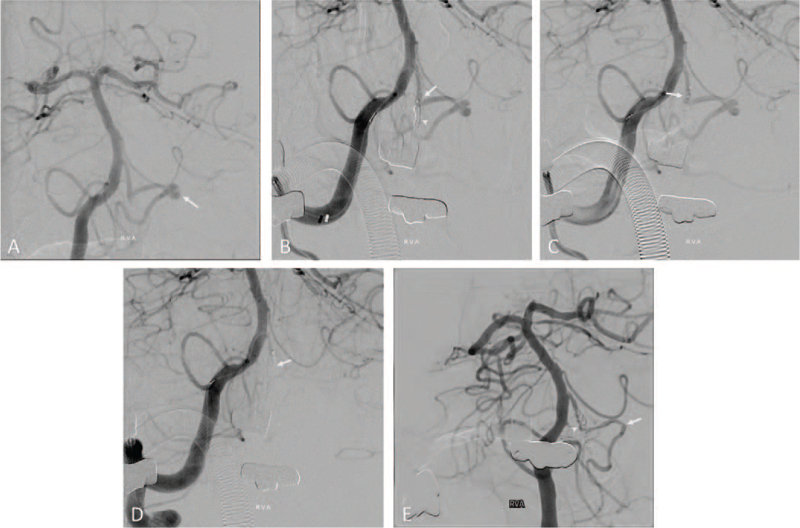
(A) Digital subtraction angiography (DSA) revealed bilobulated distal posterior inferior cerebellar artery (PICA) aneurysm (arrow). (B) During coiling embolization, we tried to reach the microcatheter into the aneurysmal sac, but it failed due to the acute angle of the vertebro-PICA junction (arrow). So, we deployed coils in the vertebral artery (VA) just before the PICA origin in order to obtain mechanical support for microcatheter navigation (arrow head). (C) Some of the coil masses deployed to VA were migrated upward of PICA origin during the navigation process using microwire and microcatheter (arrow). (D) Inevitably, we additionally deployed a coil to the VA and final DSA showed PICA occlusion (arrow). (E) Follow-up DSA after 1-year revealed recanalization of right PICA with normal configuration and aneurysmal sac on distal PICA was not seen (arrow). The recanalized VA was located medial to the deployed coil mass (arrow head).

An 80-year-old female patient presented to the emergency department of our institution with a 5-day history of headaches. A week ago, she had presented with a minor head trauma that was caused by a fall. She had a history of hypertension and dyslipidemia and had experienced focal cerebral infarction 10 years earlier. The patient had no neurologic abnormalities, except for headache. An initial non-contrast brain computed tomography (CT) scan showed a scanty subarachnoid hemorrhage (SAH) on the left ambient cistern with an enlarged ventricle (Fig. 1A). Subsequent brain CT angiography (CTA) showed no vascular abnormalities. Based on the CT findings and the patient's history of trauma, she was admitted to the neurosurgery department as a suspected case of traumatic SAH, following which her symptoms improved gradually. On the 10th day of hospitalization, the patient showed sudden stuporous mental deterioration. Brain CT performed at that time revealed SAH on all the cisterns, with intraventricular hemorrhage (Fig. 1B). A CTA performed immediately after the procedure revealed no definitive cerebral aneurysm on 3-dimensional reconstruction images, but a suspicious aneurysmal lesion was seen in the distal PICA in the thin axial CTA image (Fig. 1C). Subsequent digital subtraction angiography (DSA) revealed a bilobulated distal PICA aneurysm, and an endovascular treatment was planned (Fig. 2A). Coil embolization was performed under general anesthesia. After a right femoral artery puncture, the left vertebral artery (VA) was not visible on left subclavian angiography; therefore, a 6-Fr soft, torqueable catheter optimized for intracranial access (SOFIA; MicroVention Terumo, Tustin, CA) was advanced into the V4 segment of the right VA under roadmap guidance. Subsequently, we attempted to reach the Headway 17 microcatheter (MicroVention Terumo, Tustin, CA) into the aneurysmal sac, but it failed due to the acute angle of the vertebra–PICA junction. Therefore, after deploying the coil in the VA just before the PICA origin, we planned to position the microcatheter in the PICA with the support of the coil mass (Fig. 2B). However, some of the coil masses deployed to the VA migrated upward of the PICA origin unintentionally during the navigation process using a microwire and microcatheter (Fig. 2C). Therefore, we additionally deployed a coil to the VA and finished the procedure after confirming PICA occlusion (Fig. 2D). Fortunately, with supportive care in an intensive care unit after the procedure, the patient recovered fully, and follow-up magnetic resonance imaging showed no definitive acute cerebral infarction in the PICA territory. One year after embolization, a follow-up angiography was performed, which revealed recanalization of the right PICA with normal arterial configuration, and no aneurysmal sacs were observed in the distal PICA. The recanalized VA was located medial to the deployed coil mass (Fig. 2E).

## 4 Discussion

Herein, we report the treatment strategy and efficacy for a case of late-diagnosed distal PICA aneurysm. Although the patient's clinical outcome was good, we do not believe that we have provided the best treatment option for them; therefore, we would like to discuss the points to reflect on along with the related literature review.

First, although the patient had a history of head trauma and the amount of SAH was very small, the possibility of SAH due to aneurysmal rupture should not be excluded. When the patient first visited the emergency room, we performed a CTA, but there was a lack of detailed reviews. We were overdependent on the 3D reconstruction volume rendering images of CTA, which may have caused a delayed diagnosis of the PICA aneurysm. Usually, the PICA is extremely slender with relatively low attenuation, which makes it difficult to be visualized on 3D reconstruction images.^[3]^ Moreover, because the bone stripping technique is used to remove the bone to display the vessels alone, the PICA may only be partly visualized.^[4]^ To avoid the misdiagnosis of distal PICA aneurysms, it is essential to review the series of thin axial CTA images by adjusting the display threshold and adding the missing part of the PICA.^[5,6]^ In our case, this was not confirmed on the 3D reconstruction image of CTA images that were acquired at the time of rebleeding, but the thin axial image showed a suspicious rupture point of PICA (Fig. 1C). Although CTA is a good, less-invasive, alternative diagnostic tool, clinicians should prefer DSA—the gold standard for aneurysm detection.

Second, consideration of other treatment options and preparations for alternative strategies was insufficient when endovascular treatment did not proceed as intended. In fact, we had 2 chances to consider surgical treatment after stopping the procedure and preserving the proximal segment of the PICA—when the microcatheter did not navigate to the PICA and when the coil was deployed to the VA just proximal to the PICA origin.

Classically, the PICA can be divided into 5 segments and 2 loops based on its relationship with the medulla oblongata and cerebellum: anterior medullary, lateral medullary, tonsilomedullary, telovelotonsillar, and cortical segments, and the cranial and caudal loops.^[7]^ Therefore, PICA occlusion may be associated with 2 types of ischemic complication. First, brain stem ischemia may be caused by the occlusion of the perforating arteries supplying the brain stem, which may originate from the most proximal segments of the PICA; second, cerebellar ischemia may be caused because of distal segment occlusion of the PICA.^[8]^ Although a previous study reported that the risk of brain stem ischemia is limited because of the numerous anastomoses of the perforating arteries forming a plexiform network on the medullary surface, we should not proceed with the endovascular procedure in hopes of such good luck.^[9]^ As a result, the patient recovered well, but surgical trapping and bypass surgery were considered.

Third, prompt or short-term DSA follow-up was performed for a year after the endovascular procedure, which revealed recanalization for the normal configuration of the PICA without a visible aneurysmal sac (Fig. 2E). However, it is highly likely that the PICA flow was restored after a short period of time or immediately after the procedure. The evidence was based on the fact that the patient recovered without any neurological sequelae, and that the post-procedural MRI showed no definitive cerebral infarction. However, it is possible that the blood flow to the PICA decreased compared to that before the procedure, and that the PICA pseudoaneurysm may have regressed due to the flow diversion effect.^[10]^ The clinical course of a pseudoaneurysm is extremely unpredictable and prognosis is variable. Because all 3 layers of the artery are disrupted, rebleeding occurs easily, and delayed growth is common.^[7]^ In our patient, although the PICA pseudoaneurysm was fortunately obliterated during long-term follow-up, the aneurysm could have grown or rebleeding could have occurred if the PICA flow was maintained after the procedure.

## 5 Conclusion

Even if a patient has a history of head trauma and the amount of SAH is very small, the possibility of SAH due to aneurysmal rupture should not be excluded. When planning endovascular treatment for PICA pseudoaneurysm, an alternative option should always be considered for unexpected situations. In addition, in cases of proximal occlusion of the parent artery without internal trapping in endovascular treatment of PICA pseudoaneurysm, follow-up examination with a short-term angiogram might be crucial.

## Author contributions

**Conceptualization:** Hyung-Gyu Jang.

**Data curation:** Hyung-Gyu Jang.

**Formal analysis:** Hyung-Gyu Jang.

**Investigation:** Hyung-Gyu Jang.

**Methodology:** Hyung-Gyu Jang.

**Project administration:** Hyung-Gyu Jang.

**Resources:** Jung Soo Park.

**Software:** Jung Soo Park.

**Supervision:** Jung Soo Park.

**Validation:** Jung Soo Park.

**Visualization:** Jung Soo Park.

**Writing – original draft:** Hyung-Gyu Jang.

**Writing – review & editing:** Hyung-Gyu Jang.
